# Integrating collective know-how for multicriteria decision support in agrifood chains—application to cheesemaking

**DOI:** 10.3389/frai.2023.1145007

**Published:** 2023-04-28

**Authors:** Patrice Buche, Julien Couteaux, Julien Cufi, Sébastien Destercke, Alrick Oudot

**Affiliations:** ^1^IATE, INRAE, Univ. Montpellier, Institut Agro, Montpellier, France; ^2^I2M, INRAE, Univ. Bordeaux, Bordeaux, France; ^3^HEUDIASYC, CNRS, Univ. Compiègne, Compiègne, France

**Keywords:** ontology, decision support systems, Semantic Web, knowledge representation, expertise integration, cheesemaking

## Abstract

Agrifood chain processes are based on a multitude of knowledge, know-how and experiences forged over time. This collective expertise must be shared to improve food quality. Here we test the hypothesis that it is possible to design and implement a comprehensive methodology to create a knowledge base integrating collective expertise, while also using it to recommend technical actions required to improve food quality. The method used to test this hypothesis consists firstly in listing the functional specifications that were defined in collaboration with several partners (technical centers, vocational training schools, producers) over the course of several projects carried out in recent years. Secondly, we propose an innovative core ontology that utilizes the international languages of the Semantic Web to effectively represent knowledge in the form of decision trees. These decision trees will depict potential causal relationships between situations of interest and provide recommendations for managing them through technological actions, as well as a collective assessment of the efficiency of those actions. We show how mind map files created using mind-mapping tools are automatically translated into an RDF knowledge base using the core ontological model. Thirdly, a model to aggregate individual assessments provided by technicians and associated with technical action recommendations is proposed and evaluated. Finally, a multicriteria decision-support system (MCDSS) using the knowledge base is presented. It consists of an explanatory view allowing navigation in a decision tree and an action view for multicriteria filtering and possible side effect identification. The different types of MCDSS-delivered answers to a query expressed in the action view are explained. The MCDSS graphical user interface is presented through a real-use case. Experimental assessments have been performed and confirm that tested hypothesis is relevant.

## 1. Introduction

Agrifood chain processes are based on a multitude of knowledge, know-how and experiences forged over time. Agrifood companies that manage food product processing rely on their know-how to tailor their practices to the prevailing raw material variations, consumer expectations and regulations. The practice of acquiring knowledge through hands-on experience is a common one in the transformer industry, resulting in a vast accumulation of expertise among workers. This knowledge is typically passed on through on-the-job training and learning by doing. However, recent economic and health crises, along with internal changes within companies such as increased turnover and difficulty recruiting in certain sectors, have made it increasingly challenging to preserve and transmit this valuable know-how.

The aim of this paper, building upon the work of Buche et al. (2019), is to develop a new method for gathering and organizing knowledge, integrated in a software tool that can aid in preserving, accessing, and regularly updating the collective knowledge of the food industry for use in technology-related decision making. By implementing this methodology, we hope to overcome the challenges faced in preserving and transmitting the wealth of expertise within the industry and support the continued development of the food sector. The possibility of sustainably safeguarding and promoting practitioners' experience, as well as the technical expertise and scientific knowledge gained within a given food processing chain will be demonstrated based on a long-term collaboration with French cheesemaking companies with a “geographical indication” label, such as the protected designation of origin [*appellation d'origine protégée* (AOP)] and protected geographical indication [*indication géographique protégée* (IGP)].

The emergence of methods based on knowledge engineering in the field of food and bio-based product processing facilitates the development of decision-support tools that model complex reasoning based on processing operators' expertise (Buche et al., 2019; Baudrit et al., 2022; Belaud et al., 2022; Munch et al., 2022). Here we present a new multicriteria decision-support system (MCDSS) based on collective know-how which enables the formulation of recommendations on technological actions that may help maintain product quality or correct a product quality defect at the scale of a given food processing operation.

**Figure 1:**
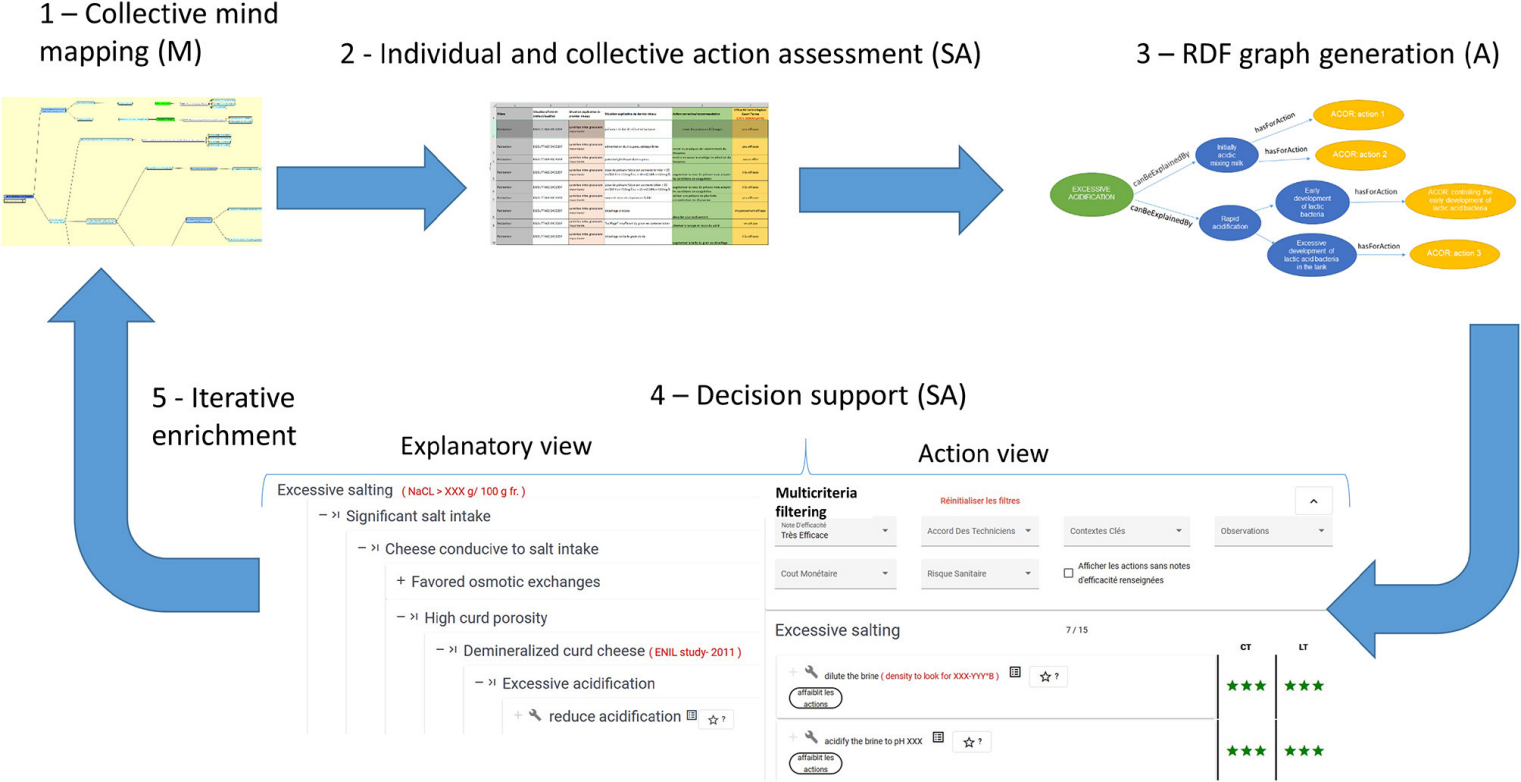
Workflow process associated with the MCDSS (M, manual task; A, automatic task; SA, semi-automatic task). The three stars indicate the action's efficiency is “very effective”.

The MCDSS workflow process presented in Figure 1 consists of five main steps. The first one is a collaborative mind mapping activity involving almost all technicians of a given food chain and coordinated by a technical expert serving as an adviser in each chain. He/she is responsible for structuring the knowledge expressed in decision trees using a mind mapping software tool that respects some simple syntactic conventions (keyword labels in nodes). One decision tree is associated with a situation of interest (a product quality or defect) while being input in a given mind-mapping file. A decision tree represents potential causal relations between the situation of interest and explanatory situations associated with recommendations in terms of technological actions to manage the situation of interest. The second step involves individually and then collectively determining the efficiency of actions based on technician feedback. This information is input in the same mind-mapping file. In the third step, the mind-mapping file is automatically translated and stored in the knowledge base implemented as an RDF knowledge graph. End-users (technicians, food chain operators, students, etc.) mine, in the fourth step, the knowledge base using two views available in the MCDSS to deliver recommendations. For a given situation of interest, the explanatory view displays all possible explanatory situations, associated analytical parameter values and technical actions to correct/reach the situation of interest. The Action View feature enables users to efficiently filter actions based on multiple criteria within a decision tree, in order to correct or reach a desired situation. Additionally, it allows users to identify any potential side effects associated with a given recommendation. Users can easily switch back and forth between the two views, facilitating the process of selecting the best recommendation for a specific situation. The MCDSS workflow process is iterative (see fifth step in Figure 1), i.e., each decision tree including action efficiency indicators may be easily updated in the mind mapping tool to account for new experiences which are then automatically translated in the MCDSS knowledge base.

The Materials and methods section focuses on the following topics:

- Specifications and architecture of the decision-support system.
- A proposed model to aggregate individual action efficiency assessments.
- An ontological model to structure MCDSS knowledge base content.
- Two views of the multicriteria decision-support system.

The Results and Discussion section presents MCDSS functionality assessments and a comparison with the current state of the art.

## 2. Materials and methods

### 2.1. Specifications and architecture of the decision-support system

The detailed MCDSS specifications were determined in collaboration with several technical centers associated with French cheesemaking, namely Comté, Reblochon, Emmental de Savoie, Cantal, and Salers in the framework of two research projects funded by the French government from 2017 to 2023 (CASDAR Docamex, France Relance Docamex). Hereafter is a list of target functionalities:

1. For a given situation of interest (targeted food quality or defect), the MCDSS must provide all known possible explanations organized in a decision tree starting from the most general explanatory situations, which must be refined by more specific explanations until it is precise enough to propose an action lever and an associated recommended technological action. It must represent interactions between explanatory situations. Two kinds of interaction should be considered: (i) conjunctive interactions of situations S_1_ and S_2_ to explain S_3_, which means that situation S_3_ may emerge only if S_1_ and S_2_ appear; (ii) strengthening (resp. weakening) interactions of situation S_1_ by situation S_2_ to explain S_3_, which means that the effect of S_1_ on S_3_ is strengthened (resp. weakened) if S_2_ appears. The decision tree will enable users to consider all possible known explanations of a given situation of interest. This functionality, which is mostly geared toward junior technicians, is very important in cheesemaking chains as they have to deal with growing turnover rates.
2. It should be possible to associate a situation (“of interest” or explanatory) with the value of a relevant analytical parameter that allows verification that the situation is actually happening. This is of great interest for technicians who have to deal with several cheese production processes (e.g., Comté and Bleu de Bresse for the CTFC technical center) without being fully aware of all of the analytical parameter values associated with the encountered situations.
3. The MCDSS must be able to determine the possible side effects of an action: a corrective action for one situation of interest should not lead another problem.
4. Feedback on technicians' individual experiences in terms of technological action efficiency to deal with a given situation of interest must be registered and aggregated. Indeed, action ranking is of great importance to help users choose the “best” action to cope with a given situation of interest. Moreover, registration of contextual criteria relevant for decision-support and associated with those assessments is required to facilitate decision support. For instance, a given action like “Review herd rationing practices” may be considered very efficient in the long term (LT), yet not at all efficient in the short term (ST). The MCDSS must be able to rank actions using a multicriteria filtering system.
5. It must represent the expert knowledge expressed in decision trees using international World Wide Web Consortium (W3C) standards in order to facilitate interoperability between industry and academic institutes in an Open Science setting. More particularly, two standard languages are recommended: (1) Resource Description Framework (RDF) for graph data description and exchange. RDF provides a variety of syntax notations and data serialization formats; (2) Web Ontology Language (OWL), a family of knowledge representation languages for authoring RDF-based ontologies.

### 2.2. From mind mapping to formal knowledge representation

Buche et al. (2019) proposed a method that enables collective mind mapping dedicated to this MCDSS. Interested readers may refer to this paper for further details on step 1 implementation (see Figure 1). In this section, we focus on two new contributions of the paper. The first concerns a numerical model that aggregates individual assessments associated with action efficiency expressed by technicians into a single indicator. This functionality is required in Specification 4 (see Section 2.1). During step 2, as presented in Figure 1, the aggregated indicator is discussed and validated collectively by the team of technicians to determine the final action efficiency value, which is input in the knowledge base for decision-making support. The second contribution is an extended version of the ontology presented in Buche et al. (2019) to structure the information in the MCDSS knowledge base for navigation and querying purposes. The extension includes the efficiency indicators and associated criteria. This extended version is expressed using the W3C standards to fulfill Specification 5 (see Section 2.1), which is also a novel contribution of this paper as the ontology presented in Buche et al. (2019) was based on the Conceptual Graph model (Sowa, 1984; Chein and Mugnier, 2009).

#### 2.2.1. A model to aggregate individual action efficiency assessments

Each technician, denoted *T*_*i*_ hereafter, provides two types of information:

- His/her experience in terms of number of action implementations, called *F*_*i*_, reflecting the reliability of his/her statements, which takes its value in the set {(*N*)ever, (*R*)arely: 1 < 3, (*S*)ometimes: 3 < 10, (*O*)ften): > 10}, as summarized by *R* = {*N, R, S, O*}.
- The efficiency of the action, denoted *E*_*i*_, which takes its value in {Very effective (*A*), Moderately effective (*B*), Not very effective (*C*), No effect (*D*)}, as summarized by *E* = {*A, B, C, D*}.

The technician can also select “don't know” for the second value.

With the experience of the technician corresponding to the number of times (roughly) where he/she encountered the situation of interest, it seems quite natural to interpret his/her answer as a number of “virtual” observations. We will therefore associate with each value in *R* an equivalent number, i.e., *N* → *0.5, R* → *2, S* → *5, O* → *10*. In practice, each of these values is chosen to be within the corresponding interval. For instance, *Rarely* corresponds to the interval [1,3], for which we picked the central value 2. We still assigned a positive value to *Never*, so as to reflect the fact that the reported experience may come from sources other than direct observation. Those choices were made in accordance with the end user and can in practice be changed according to the application, as they remain subjective (but not arbitrary) to some extent. The corresponding intervals could in principle also be kept, yet processing such information would increase the cognitive load for users, hence our choice to keep precise numbers representing the numbers of experiments.

Let *n*_*i*_ be the number corresponding to the experience of technician *T*_*i*_. For example, if technician *T*_*i*_ answers *F*_*i*_ = *R*, therefore rarely, then *n*_*i*_ = 2. If *k* technicians provide an answer, then $total\;N=\overset{k}{\underset{i=1}{\sum }}n_{k}$ will denote the total number of virtual observations.

The aim is then—based on these virtual observations—to construct a histogram on *E*, and associate a probability with each of its elements. Let *n*^*A*^, *n*^*B*^, *n*^*C*^, *n*^*D*^ denote the total number of observations given to *A, B, C, D*, respectively.

**Definition 1:** *n*^*A*^ the total number of observations given to *A* is defined by

$$\begin{array}{l} n^{A}=\underset{T_{i}:\;E_{i}=A}{\sum }n_{i}\; \end{array}$$

The probability (subjective and *a priori*) of *A* then becomes

$$\begin{array}{l} p\left(A\right)=\frac{n^{A}}{Nb}\; \end{array}$$

and the same for *B, C, D*.

**Example 1:** Suppose three technicians provide their opinions as follows:

- *F*_1_ = *R* ⇒ *n*_1_ = 2; *E*_1_ = *A* (very effective)
- *F*_2_ = *S* ⇒ *n*_2_ = 5; *E*_2_ = *B* (moderately effective)
- *F*_3_ = *R* ⇒ *n*_3_ = 2; *E*_3_ = *C* (not very effective)

which gives *N* = 9 and *p(A)* = 2/9; *p(B)* = 5/9; *p(C)* = 2/9.

The information given by the previous distribution is probably too complex to be readily understood by a technician and requires a simple summary. This can easily be done through various statistics and then supplied to the user in graphical and easily interpretable form. In contrast with number of times a situation has been encountered, in our case efficiency is not associated with an actual numerical measure. Moreover, such measures would probably vary across situations and not be comparable. We therefore chose to not replace ordered categories A, B, C by numbers, and instead provided both a central value and its dispersion based on the quantile notion. More precisely, we will use the median (quantile at 50%) and two quantiles around the latter (therefore 50% – α and 50% + α) as a statistical summary.

**Definition 2:** the quantile of level ∈ [0, 1], denoted *i*_β_, relative to the distribution *p* defined on *E* is the value

$$\begin{array}{l} i_{\beta }=\left\{j\in E:\;\left(\underset{l\leq j-1}{\sum }p\left(l\right)<\beta \right)\wedge \left(\underset{l\leq j}{\sum }p\left(l\right)\geq \beta \right)\right\}\; \end{array}$$

where < corresponds to the alphabetical order and with the convention $\underset{l\leq 0}{\sum }p\left(l\right)=\;0$.

Let us get back to our previous example, where we will conventionally denote *P*({*A, B*}) = *p*(*A*) + *p*(*B*), etc.

**Example 2-1:**

P({A}) = p(A) = 2/9 = 0.2222.

P({A,B}) = p(A) + p(B) = 2/9 + 5/9 = 0.77777.

P{A,B,C}) = P({A,B,C,D}) = 1.

We will therefore have the following quantile *i*_0.1_ = *A* (first decile) because $\underset{l\leq 0}{\sum }p\left(l\right)=0$ and $\underset{l\leq A}{\sum }p\left(l\right)=0.2222$ therefore$\left\{\left(\underset{l\leq 0}{\sum }p\left(l\right)<0.1\right)\wedge \left(\underset{l\leq A}{\sum }p\left(l\right)\geq 0.1\right)\right\}$ is true.

In the same way, *i*_0.25_ = *B* (first quartile); *i*_0.5_ = *B* (median); *i*_0.75_ = *B* (third quartile); *i*_0.9_ = *C* (ninth decile).

It is clear that if the technicians all provide the same evaluation, then all the quantiles will have the value of this evaluation. Conversely, if the technicians are somewhat divided and of equivalent experience, the difference between the quantiles will show this uncertainty. We hence propose to match A, B, C, D to a number of “stars” (3,2,1,0) and to provide the average of the values observed in set [*i*_0.1_, *i*_0.9_] *as a reference value*. In our example, this is the set [A, B, C], with the reference value 2. It would also be useful to show that there is no consensus on this reference value by highlighting all the intervals [1,3].

**Figure 7** presents examples of graphical representations in terms of stars. The following example illustrates the case where the reference value is not one of the initial values.

**Example 2-2**:

Suppose that two technicians provide their opinions as follows:

- *F*_1_ = *R* ⇒ *n*_1_ = 2; *E*_1_ = *A* (very effective)
- *F*_2_ = *P* ⇒ *n*_2_ = 5; *E*_2_ = *B* (moderately effective)

In this case, p(A) = 2/7, p(B) = 5/7 with *i*_0.5_ = *B* (median) and [*i*_0.1_, *i*_0.9_] = [*A, B*] with 2.5 being the obtained average (stars).

#### 2.2.2. A new ontological model to structure the MCDSS knowledge base content

Decision trees edited in mind-map files in step 1 and enriched with action efficiency assessments in step 2 must be stored in the MCDSS knowledge base. As indicated in Specification 5, Semantic Web language standards created by W3C must be used for knowledge base implementation. The OWL ontology—an original contribution of this paper—designed to structure and instantiate a decision tree in the RDF knowledge base is presented in this section.

**Figure 2:**
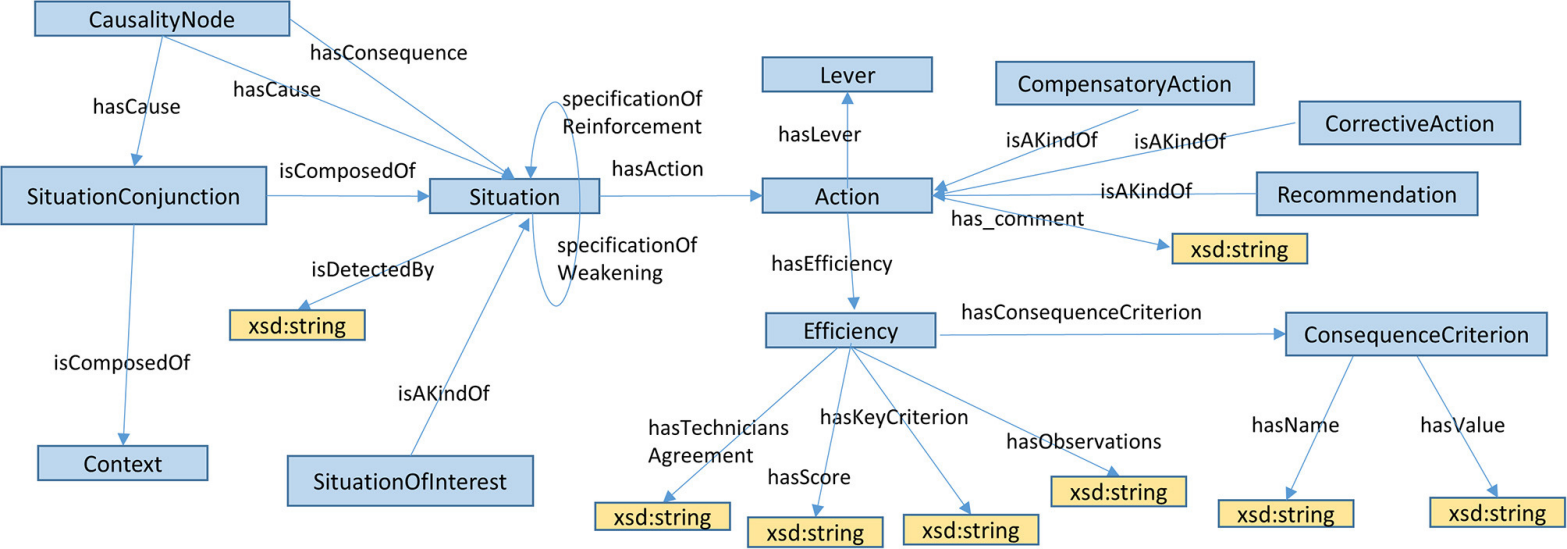
OWL ontological model used to structure a decision tree in the MCDSS knowledge base.

The OWL definition of classes and properties presented in Figure 2 is available in Buche et al. (2022). Hereafter we explain how this ontological model takes the specifications expressed in Section 2.1 into account.

As expressed in Specification 1, a situation S_1_, an instance of the *Situation* OWL class, is explained by a situation S_2_ through an instance of the *CausalityNode* class linked to S_1_ (resp. S_2_) by the OWL *hasForCause* (resp. *hasForConsequence*) object property. Note that S_1_ may be an instance of the *SituationOfInterest* class that is a kind of *Situation*. A situation S may be associated with an action A via the *hasForAction* object property. An action A is associated with its lever through the *hasForLever* object property. A conjunctive interaction CI_1_, an instance of the *SituationConjunction* class, is linked to conjunctive causal situations S_1_ and S_2_ (and other situations if required) by the *isComposedOf* property. CI_1_ is linked to an instance of the *CausalityNode* class by the *hasForCause* property. This CausalityNode class instance is linked to the consequence situation S_3_ by the *hasForConsequence* property. The strengthening (resp. weakening) interaction of situation S_1_ by situation S_2_ to explain S_3_ is also represented using a conjunctive interaction CI_1_, an instance of the *SituationConjunction* class. The asymmetric role of situations is achieved in the following way: the altered situation S_1_ is linked to altering situation S_2_ via the *SpecificationOfWeakening (resp. SpecificationOfReinforcement)* object property if the alteration type is weakening (resp. strengthening).

The *isDetectedBy* datatype property associated with an instance of the *Situation* class implements Specification 2. An instance of *Action* is associated with an instance of the *Efficiency* class to implement Specification 4. The *hasForKeyCriterion* datatype property permits determination of the list of criteria values associated with a single *Efficiency* instance. The *hasForScore, hasForObservations*, and *hasForTechniciansAgreement* datatype properties are associated with an *Efficiency* class instance which is linked to an *Action* instance. The *hasForConsequenceCriterion* object property links an *Efficiency* instance with a set of pairs (name, value) that are used for decision support. The *refersToDefect* object property links an *Efficiency* instance with the situation of interest to which it refers.

**Figure 3:**
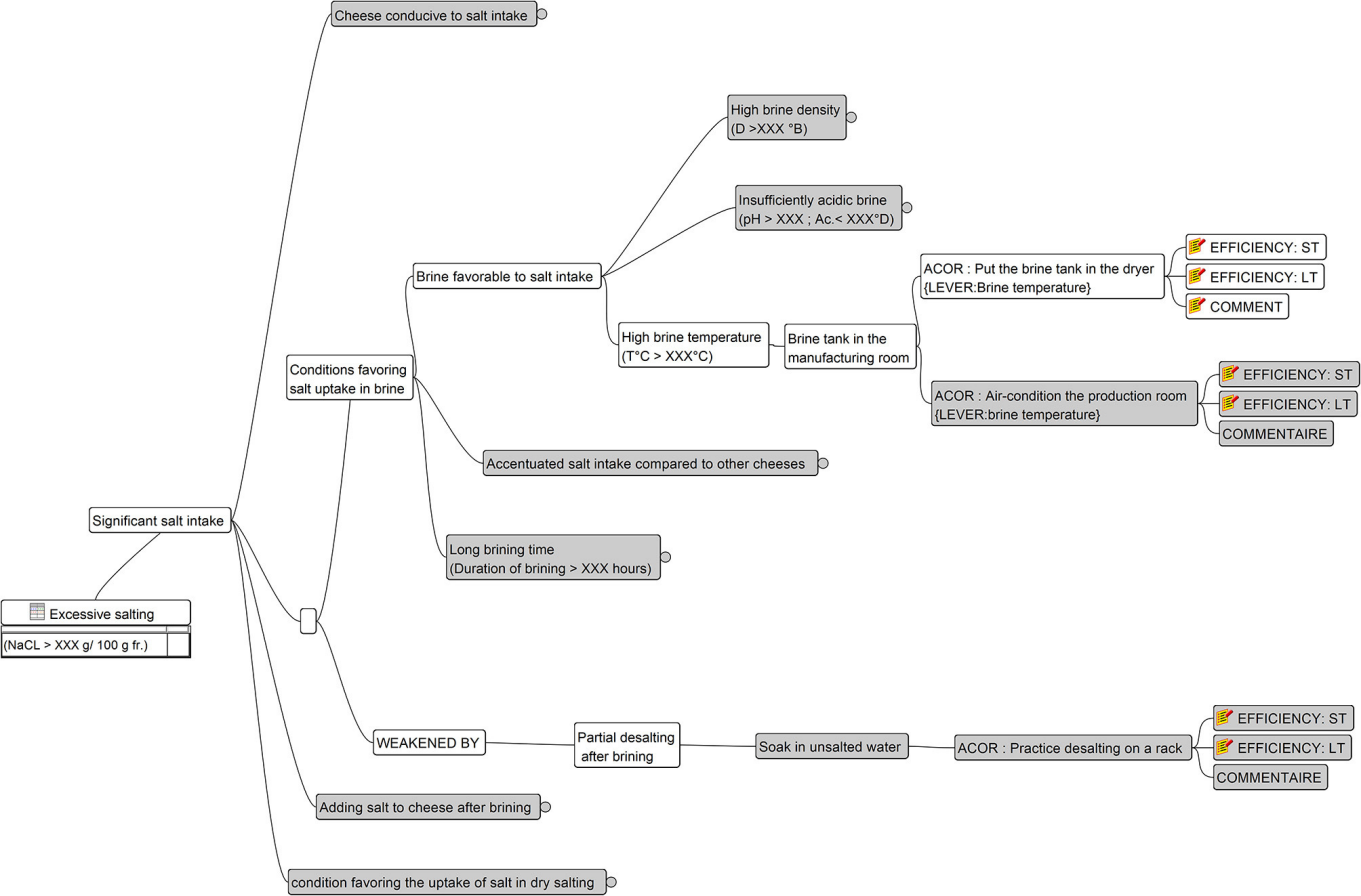
An excerpt of the mind-mapping file associated with the *Excessive salting* situation of interest.

**Figure 4:**
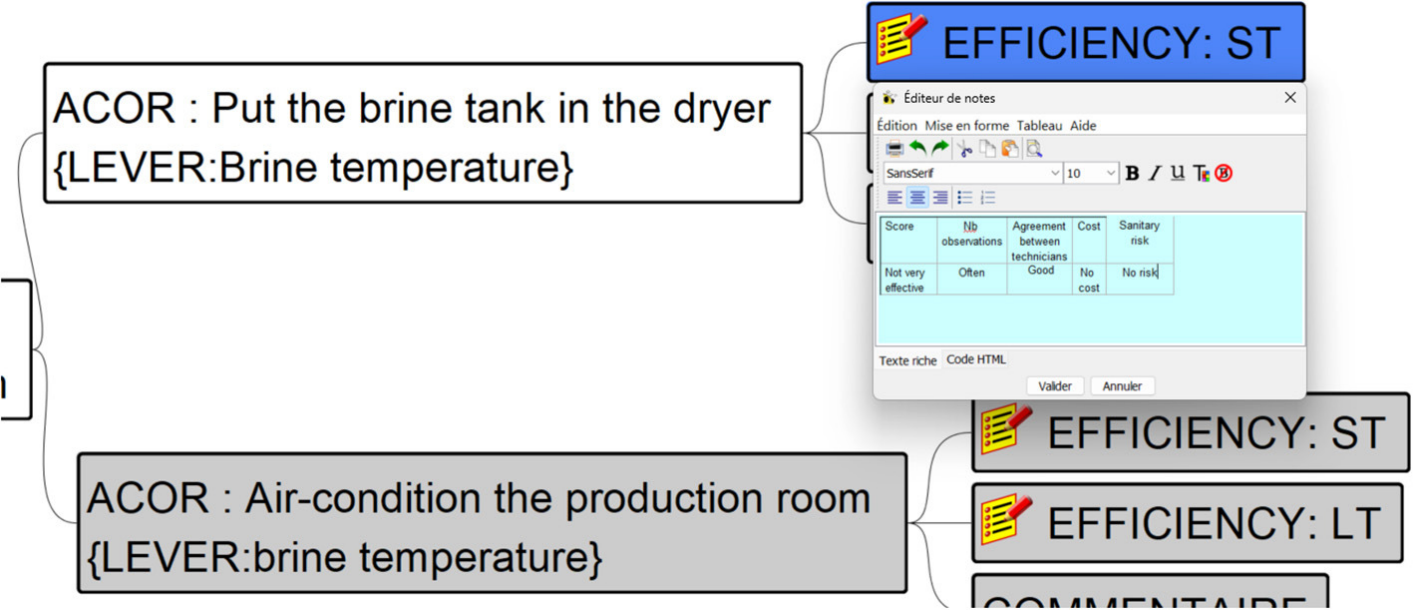
Zoom on the table associated with the EFFICIENCY: ST node present in the mind-mapping file associated with the *Put the brine tank in the dryer* node.

**Figure 5:**
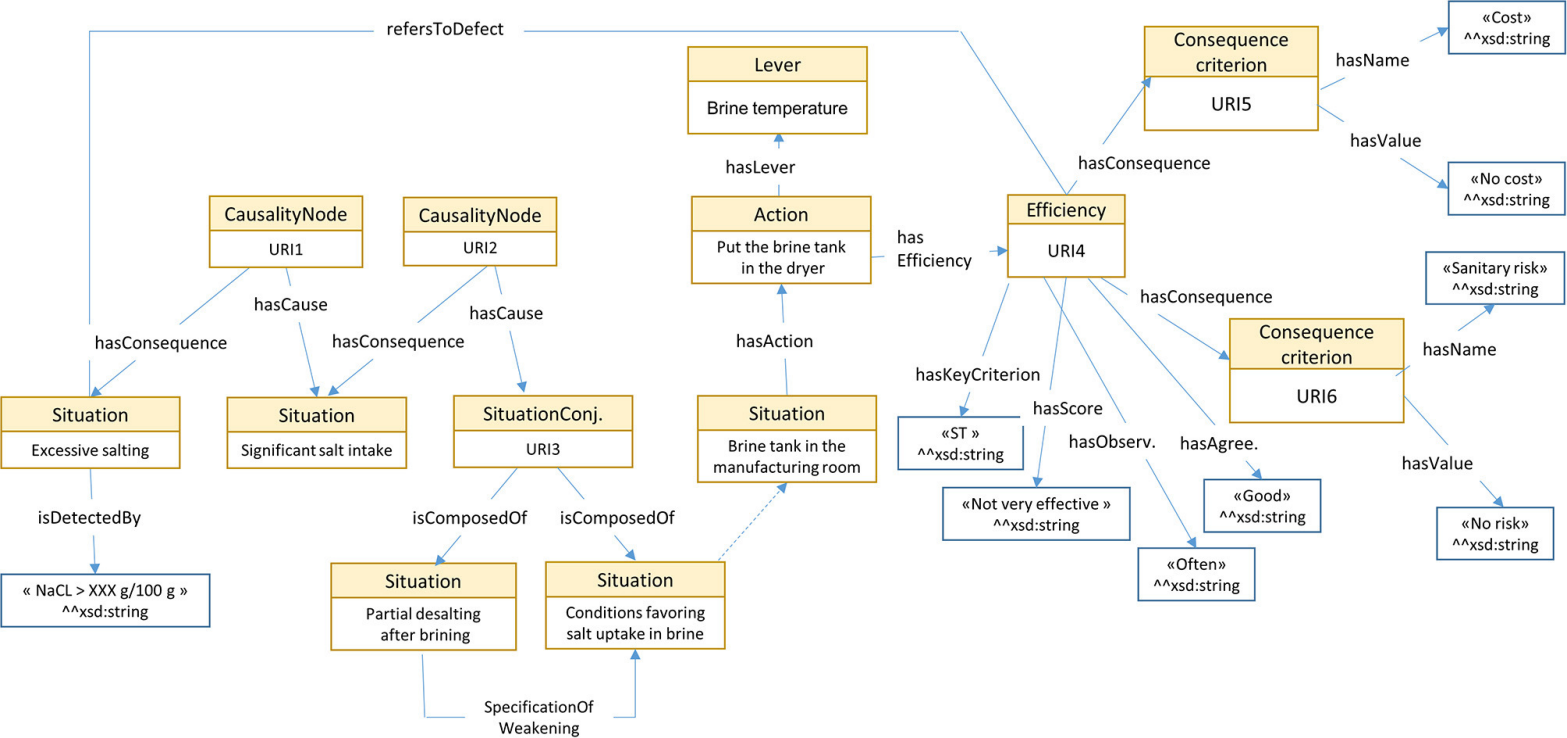
An excerpt of the MCDSS knowledge base corresponding to the selected branch in Figure 3.

Figure 3 is an excerpt of a mind-mapping file representing the decision tree associated with the situation of interest *Excessive salting* achieved by the blue node at the bottom left part of the figure. The entire mind-mapping file is available in Buche et al. (2022). This situation of interest may be explained by the *Significant salt intake* situation. Then four explanations are possible. Hereafter we will consider the one whose node is white, i.e., *Conditions favoring salt uptake in brine* and its associated branch, whose nodes are also white, until reaching the two nodes *Put the brine tank in the dryer* and EFFICIENCY: ST. Figure 4 shows a zoom on the table associated with the node EFFICIENCY: ST. This table includes the aggregated efficiency indicator with the number of observations (see Section 2.2.1) and contextual criteria associated with them. In Figure 5, we present a part of the MCDSS RDF knowledge base corresponding to the translation of the branch whose nodes are white in the decision tree presented in Figure 3. The entire RDF graph corresponding to the mind-mapping file is available in Buche et al. (2022).

In Figure 5, to facilitate the understanding of the translation of Figures 3, 4 into RDF, instances of OWL classes are represented by rectangles, with the class name in the header complemented by a pseudo-label representing its URI (as the real one is too long) or the associated value of the rdfs:label property. Values associated with datatype properties are framed in black.

### 2.3. Multicriteria decision-support system

The decision-support system (see step 4 in Figure 1) consists of two complementary access modes to the knowledge base content, i.e., the explanatory and action views. The explanatory view displays the decision tree associated with a given situation of interest, including all possible explanatory situations, associated analytical parameter values and technical actions to correct/reach the situation of interest. The action view displays the list of actions related to a given decision tree to correct/reach the associated situation of interest. It enables multicriteria filtering, action ranking and side effect identification.

Both views may be used independently and jointly depending on the usage case. For instance:

- A systematic review of all possible explanatory situations is carried out using the explanatory view.
- Solving a contextualized problem is carried out using the action view through the multicriteria filtering mode, sometimes complemented with the explanatory view.

Hereafter we define a multicriteria (MCDSS) query executed in the action view and the associated answers. Then we present the MCDSS graphical user interface (GUI) using an illustrative example based on a real case from a French protected designation of origin (AOP) chain.

#### 2.3.1. MCDSS query definition

We define in this section the notion of MCDSS query *Q* executed on the *KB* knowledge base. Then the answer to *Q*, called *AN*, and the two complementary *AN-inter* and *AN-intra* answers are defined for side effect identification.

**Definition 3:** The MCDSS knowledge base (*KB*) is defined as the 9-tuple (*S*, *V*_*k*_, *C*_*c*_, *V*_*c*_*, A, L, E, Ag, O*), with:

- *S* = the set of instances of the *SituationOfInterest* class;
- *V*_*k*_ = the set of key criteria labels associated with the *hasForKeyCriterion* datatype property;
- *C*_*c*_ = the set of consequence criteria names associated with the *hasForName* datatype property;
- *V*_*c*_ = the set of consequence criteria values associated with the *hasForValue* datatype property;
- *A* = the set of *Action* class instances implemented using *Lever* class instances;
- *L* = the set of *Lever* class instances;
- *E* = the set of action efficiency labels associated with the *hasForScore* datatype property;
- *Ag* = the set of action efficiency consensus labels associated with the *hasForTechnicianAgreement* datatype property;
- *O* = the set of action efficiency labels associated with the *hasForObservations* datatype property.

**Definition 4:** Given *KB* defined in Def. 3, the set of input conjunctive filtering parameters associated with an MCDSS query *Q* executed in *KB* is defined by the 6-tuple:

$$\begin{array}{l} (s\in S,\;\left\{v_{1},\;\cdots \;,v_{m}\right\}\;\in V_{k},\;\left\{\left(c_{1},v_{1}\right),\cdots \;,\left(c_{n},v_{n}\right)\right\}\epsilon {\left(C_{c},V_{c}\right)}^{n},\;\\ \left\{e_{1},\;\cdots \;,e_{o}\right\}\;\in E,\;\left\{ag_{1},\;\cdots \;,ag_{p}\right\}\;\in Ag,\;\left\{o_{1},\;\cdots \;,o_{q}\right\}\;\in O) \end{array}$$

Note: multivalued parameters are considered to be aggregated disjunctively in the querying.

**Example 3:** *Q1* = (*excessive salting*,{∅},{∅}, {*very effective*}, {*good, average*}, {∅}) represents the querying of the *excessive salting* situation of interest with the action efficiency being *very effective* and the action efficiency consensus being *good* or *average*. The SPARQL query generated by the MCDSS and corresponding to *i*s available in Buche et al. (2022).

**Definition 5:** The answer *AN* associated with an MCDSS query *Q* executed in *KB* is defined by a set of 2-tuples: $\left\{\left(a_{1},l_{1}\right),\cdots \;,\left(a_{n},l_{n}\right)\epsilon {\left(A,L\right)}^{n}\right\},\;$ with (*a*_*i*_, *l*_*i*_) related to the decision tree associated with the situation of interest *s*.

**Example 4:** *AN1* = {*(dilute the brine, Brine salt concentration), (acidify the brine to pH 5.4, Brine acidity), (practice brining on a rack, Brining equipment), (reduce brining time, Brine duration)*} is the answer that includes the four recommended actions associated with the *q*uery of Example 1. The triples results of the SPARQL query corresponding to *i*s available in Buche et al. (2022).

Two complementary answers with *AN* are provided by the MCDSS when the *Q* query is executed. The objective, corresponding to Specification 3 (see Section 2.1), is to identify two types of potential side effects that could occur if a recommended action related to *AN* is implemented:

- *AN-inter*: potential side effects with other situations of interest related to *KB*. Situations where the associated decision tree recommends the use of a lever associated with a given *AN* action are selected.
- *AN-intra*: potential side effects with other actions related to the decision tree associated with the situation of interest expressed in *Q*.

**Definition 6:** Given the *AN* answer to a query *Q*, the *AN-inter* answer associated with a recommended *a*_*i*_ *action* implemented using a given *l*_*i*_ lever with (*a*_*i*_, *l*_*i*_) ∈ *AN* is defined by a set of 2-tuples:

($s^{\prime }\in S,\;\left\{\left(a_{1},l_{i}\right),\cdots \;,\left(a_{n},l_{i}\right)\epsilon {\left(A,L\right)}^{n}\right\})$ with *s*′ ≠ *s*, with s being the situation of interest associated with the *Q* query and (*a*_*j*_, *l*_*i*_), *j* =*1, …n* being related to the decision tree associated with the *s'* situation of interest.

**Example 5:** *AN-inter1* associated with the recommendation *(reduce brining time, Brine duration)* related to *AN1* is *{(unpleasant taste or odor*, {(*extend the brining time to 2 h maximum, Brine duration)}),(brown paste*,{(*extend the brining time to 2 h maximum, Brine duration)}), (excessive proteolysis*,{(*extend the brining time to 2 h maximum, Brine duration)}), (insufficient salting*, {(*extend the brining time to 2 h maximum, Brine duration)})}*.

*AN-inter1* means that implementing the recommendation *reduce brining time* to solve the *excessive salting* situation may create a side effect with four other situations of interest likely to occur: *unpleasant taste or odor, brown paste, excessive proteolysis, insufficient salting*. Indeed, the same *Brine duration* lever is recommended to solve these situations but it is used in an opposite way *(extend the brining time to 2 h maximum)*, which could potentially trigger those situations of interest if the recommendation is applied. MCDSS users may query the decision trees associated with those situations of interest to find a good trade-off to avoid triggering unwanted side effects.

**Definition 7:** The *AN-intra* answer associated with an *a recommended action* implemented using a given *l* lever to solve the *s* ∈ *S* situation of interest is defined by a 4-tuple:

($\left\{\left(a_{11},l_{1}\right),\cdots \;,\left(a_{1n},l_{n}\right)\right\}\epsilon {\left(A,L\right)}^{n}\},\;\left\{\left(a_{21},l_{1}\right),\cdots \;,\left(a_{2n},l_{n}\right)\right\}\epsilon$${\left(A,L\right)}^{n}\},\;\left\{\left(a_{31},l_{1}\right),\cdots \;,\left(a_{3n},l_{n}\right)\right\}\epsilon {\left(A,L\right)}^{n}\},\;\{\left(a_{41},l_{1}\right),\cdots \;,$$\left(a_{4n},l_{n}\right)\}\epsilon {\left(A,L\right)}^{n}\})$ with *a*_1*i*_ actions (resp. *a*_2*i*_ actions) corresponding to potential weakening actions of the recommended *a* action (resp. potential actions weakened by the recommended *a* action) and *a*_3*i*_ actions (resp. *a*_4*i*_ actions) corresponding to potential reinforcement actions of the recommended *a* action (resp. potential actions reinforced by recommended *a* action).

**Example 6:** *AN-intra1* associated with the recommendation *(reduce brining time, Brine duration)* related to *AN1* is *({(practice desalting on a rack, Brining equipment*)*)}),{*∅*},{*∅*}{*∅*})*.

AN-intra1 means that implementing the *reduce brining time* recommendation to solve the *excessive salting* situation may be weakened by the *practice desalting on a rack* action. Complementary information about this possible interaction may be found using the explanatory view.

#### 2.3.2. MCDSS graphical user interface

Using an illustrative example, we show how the MCDSS graphical user interface has been implemented to propose both complementary access modes to the knowledge base content, i.e., the explanatory and action views.

**Figure 6:**
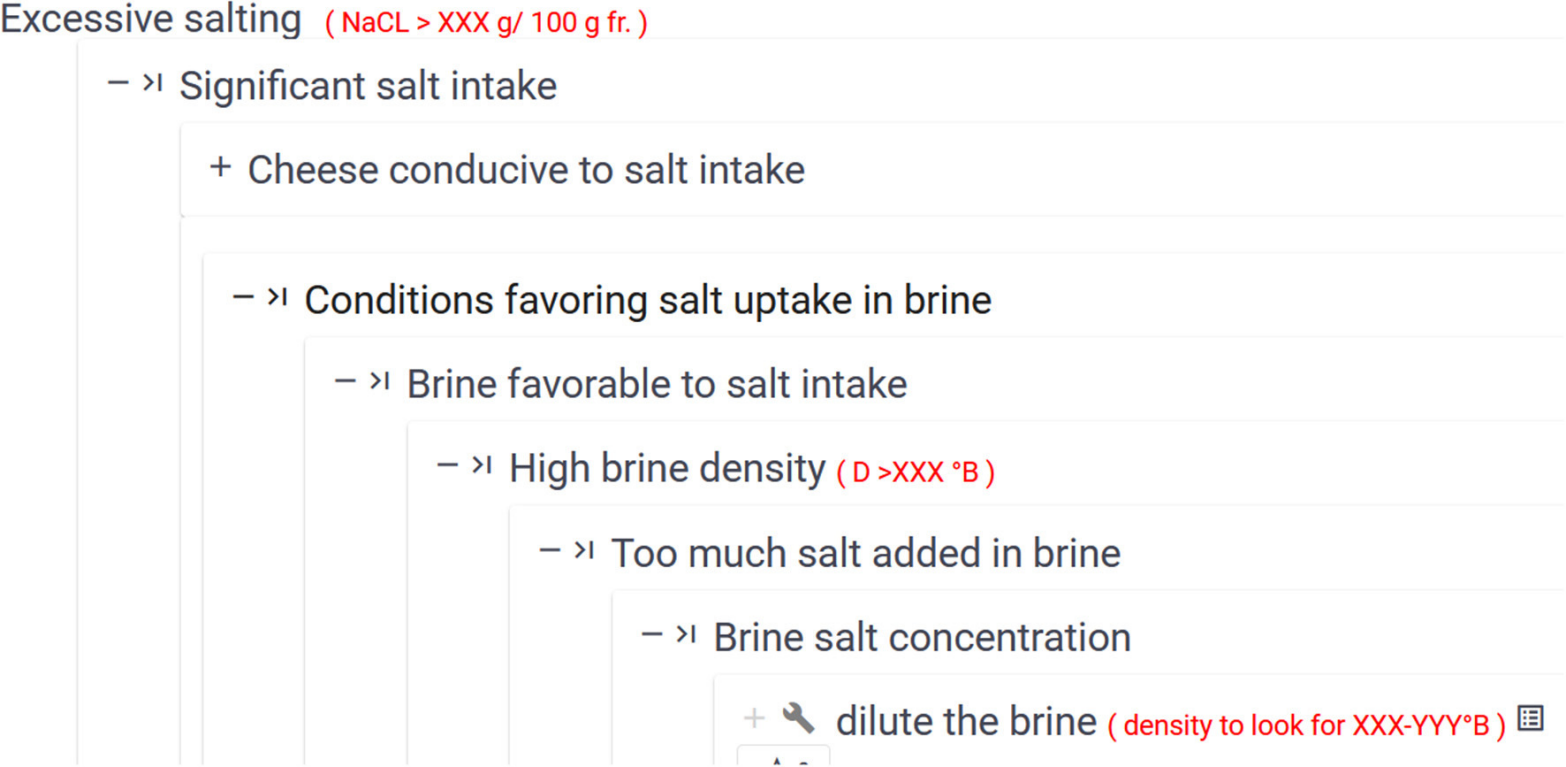
MCDSS explanatory view showing an excerpt of the decision tree associated with the *Excessive salting* situation of interest.

The explanatory view proposes navigation in a decision tree associated with a given situation of interest to query all possible explanatory situations. Figure 6 shows an excerpt of the explanatory view for the *Excessive salting* situation of interest. Analytical values associated with situations are shown in red. For example, *NaCl rate* > *XXX* ^1^*g/100g* is the value associated with the *Excessive salting* situation. The first high-level explanatory situation is *Significant salt intake by the cheese during its production*, while several others specify this high-level explanation. For instance, it could be explained by the *Conditions favoring salt uptake in brine* situation. By following this branch of the decision tree, we reach a more detailed explanation, i.e., *Too much salt added in brine*. This latter explanation is associated with the *Dilute the brine* action. Its associated analytical value is *Density to reach XXX-YYY*° *B*.

**Figure 7:**
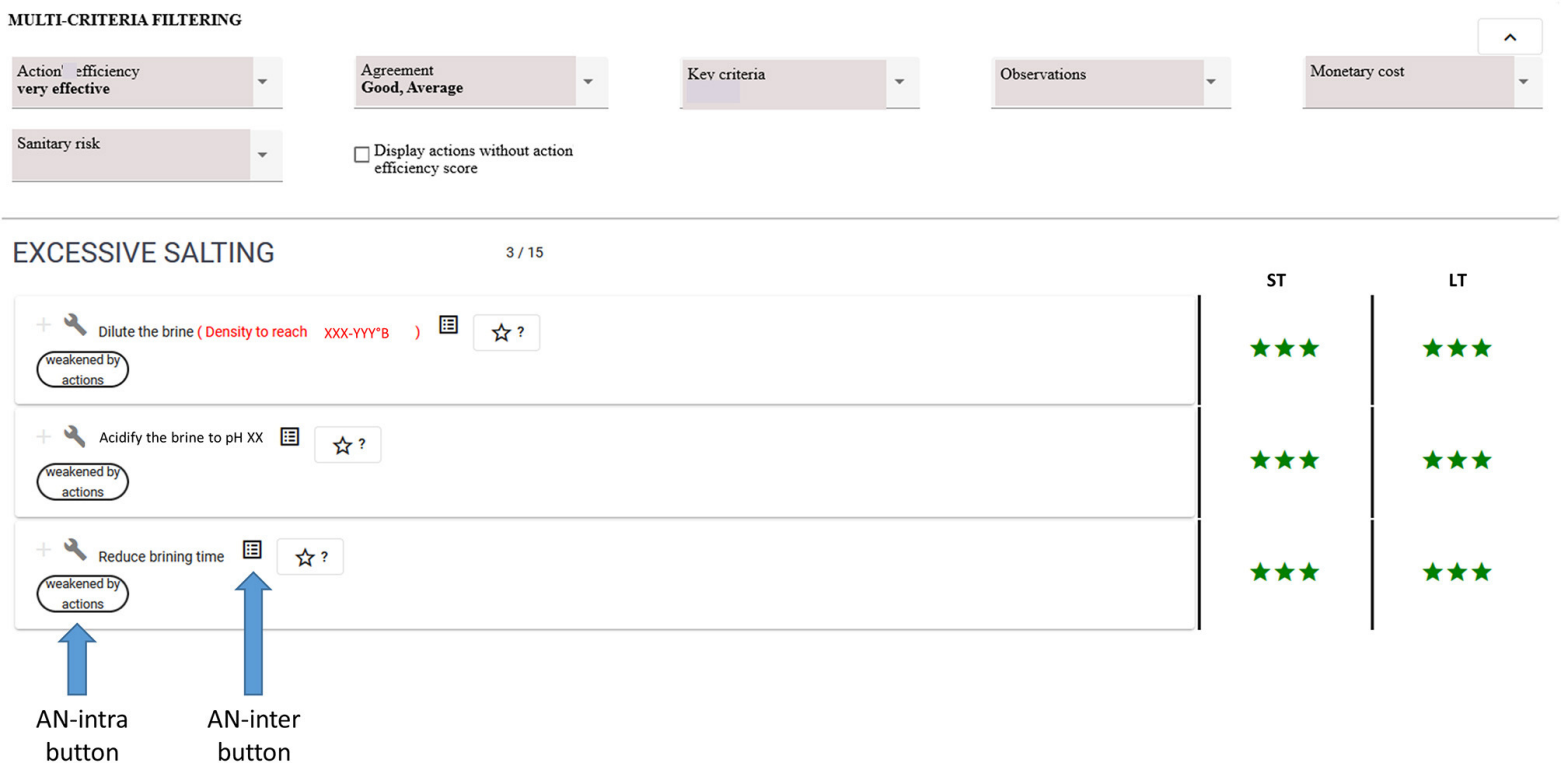
MCDSS action view showing an excerpt of the list of filtered actions related to the decision tree associated with the *Excessive salting* situation of interest. The three stars indicate the action's efficiency is “very effective”.

The action view enables knowledge base querying and filtering to solve a contextualized problem. Figure 7 shows a query presented in example 3 concerning the *Excessive salting* situation of interest. Filtering criteria used regarding the action efficiency indicator and agreement level enable filtering of three actions out of a total of 15 present in the decision tree.

**Figure 8:**
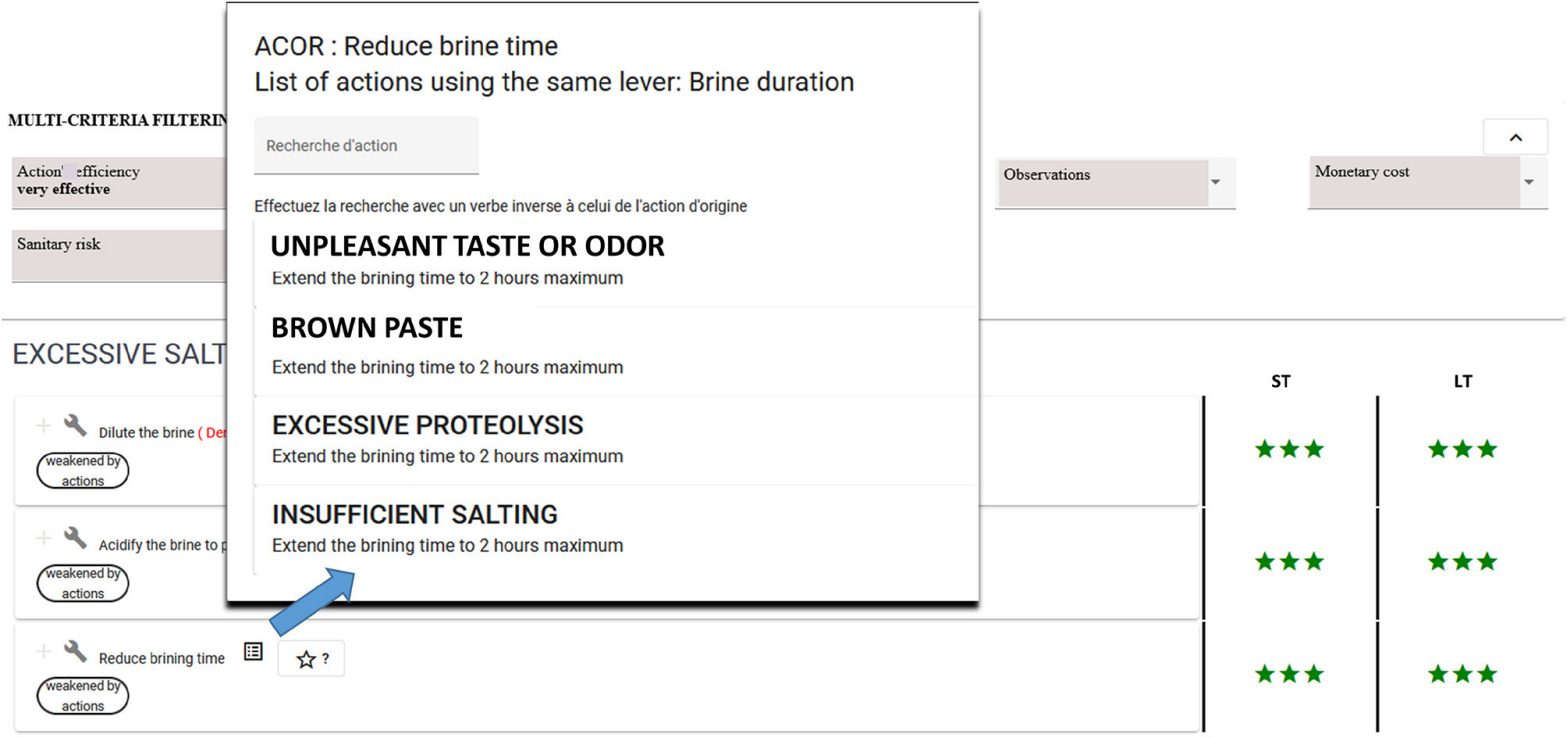
MCDSS action view showing a list of four situations of interest using the same lever but in an opposite way compared to that recommended to solve the *Excessive salting* situation of interest. The three stars indicate the action's efficiency is “very effective”.

**Figure 9:**
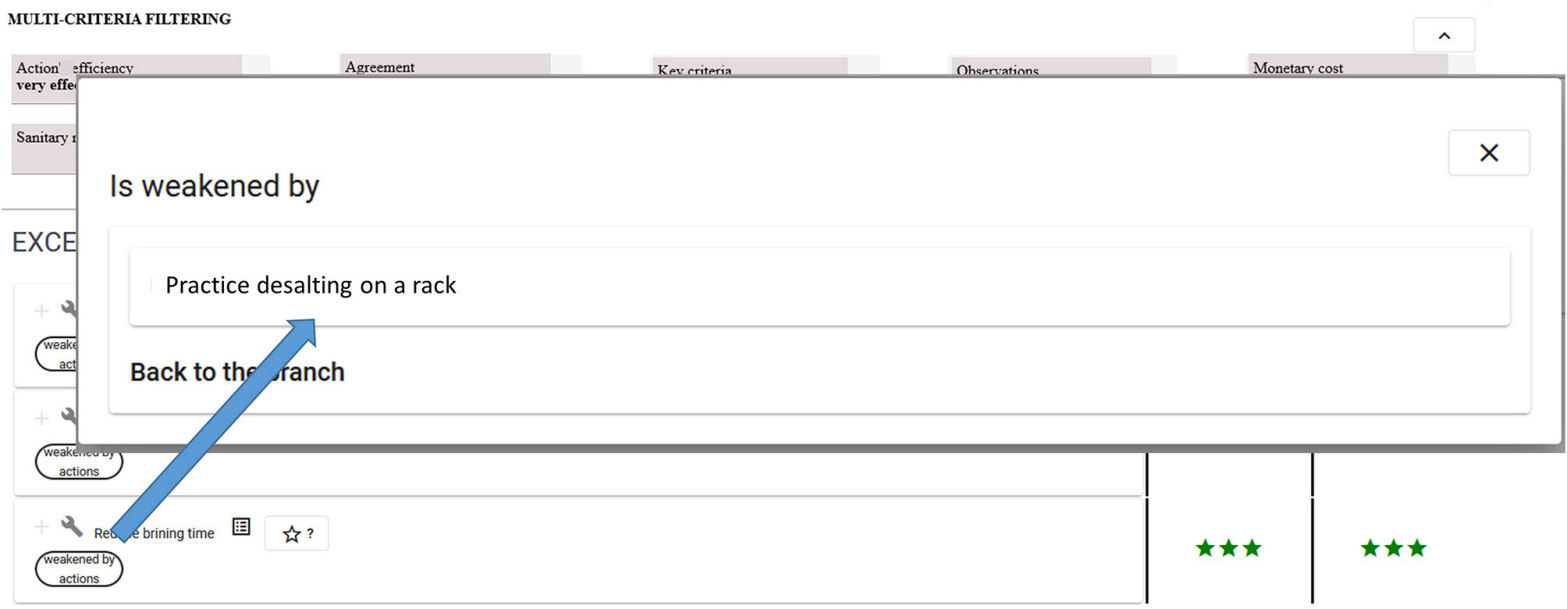
MCDSS action view showing a list of actions related to the decision tree associated with the *Excessive salting* situation of interest which could potentially weaken the recommended action. The three stars indicate the action's efficiency is “very effective”.

Complementary answers identifying possible side effects may be obtained using buttons (see the two buttons at the bottom of Figure 7 corresponding to AN-intra and AN-inter answers for the *Reduce brining time* action). Figure 8 shows a list of four situations of interest presented in Example 5 above: *unpleasant taste or odor, brown paste, excessive proteolysis*, and *insufficient salting*. The *Brine duration* lever is recommended to solve these situations, while using it in an opposite way *(extend the brining time to 2 h maximum)* compared to that recommended for the *Excessive salting* situation of interest. Figure 9 shows the action presented in Example 5, which may weaken the recommended *Reduce brining time* action.

## 3. Results

In this section, we present the assessment results of Specifications 1, 2, and 4, which were performed with end-users.

### 3.1. Reviewing all possible technological actions associated with a situation of interest (Specifications 1 and 2)

**Figure 10:**
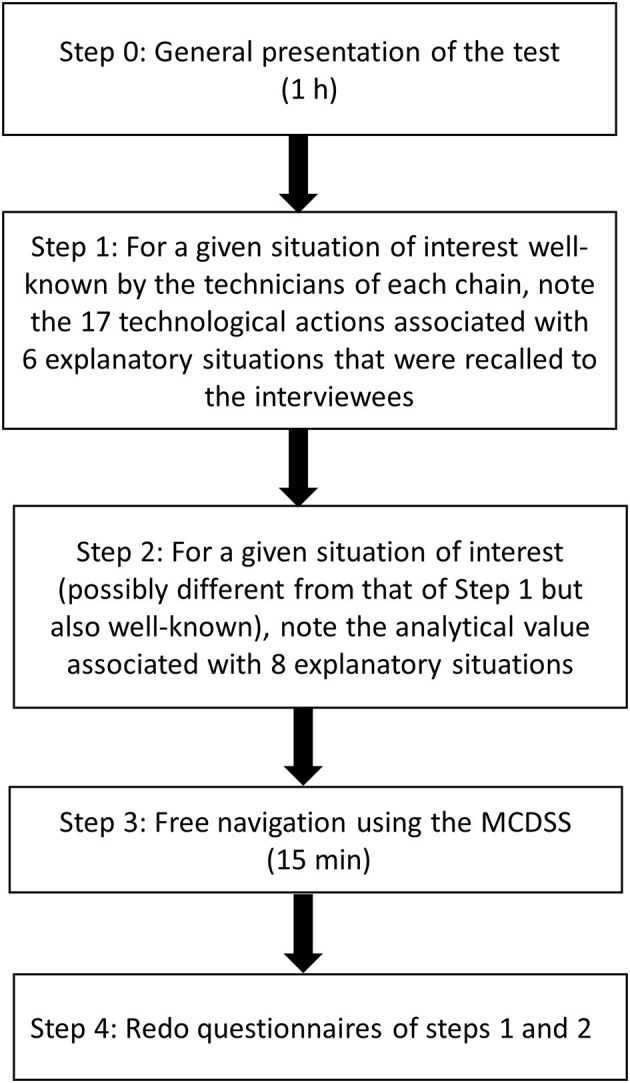
Protocol designed to assess the impact of MCDSS use in the reasoning task.

**Figure 11:**
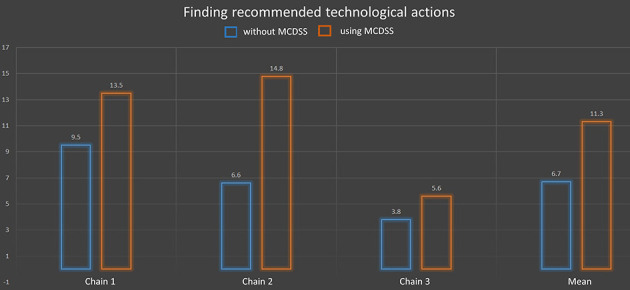
Test results (blue without MCDSS, brown with MCDSS) for recommended technological action findings: the y-axis represents the number of correct answers (mean value) in the three chains and on average.

**Figure 12:**
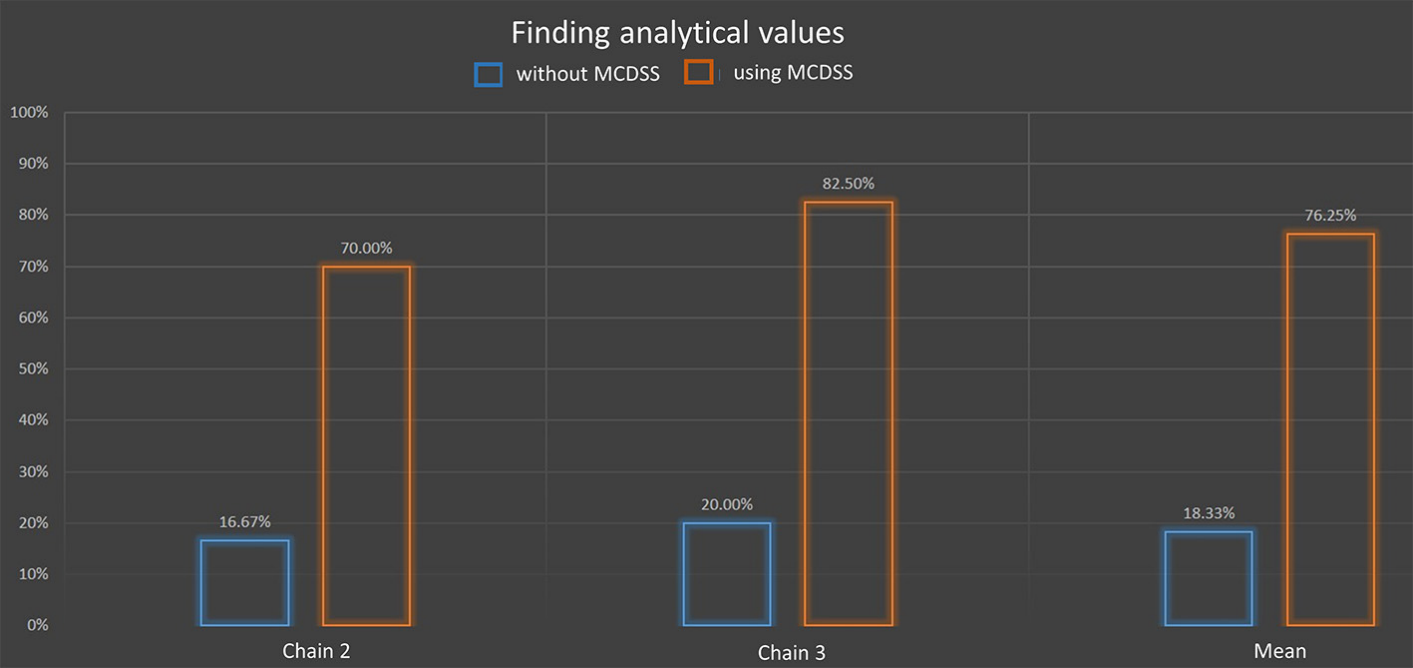
Test results (blue without MCDSS, brown with MCDSS) for analytical value findings: the y-axis represents the percentage of correct answers (mean value) in two chains and on average.

In a technological reasoning task, for a given situation of interest (targeted quality or defect), a technician must be able to check all possible explanatory situations and corresponding analytical parameters to check that this situation will happen. Moreover, he/she must be aware of the associated recommended technological action. The protocol presented in Figure 10 was designed to assess the impact of MCDSS use in this reasoning task. It was tested with technicians from three different food chains. The protocol includes the following steps:

- Fifteen people related to three different chains passed this test. Figure 11 shows that 39% of the technological actions were noted without the MCDSS and 66.5% after its use, which represents 27% enhancement. Only two chains (10 people) undertook the analytical value tests as chain 1 corresponds to generic knowledge associated with a situation of interest learned in technical schools. As analytical values highly depend on a given cheesemaking process, it was not possible to conduct this test on this generic knowledge. Figure 12 shows that 18.33% of the correct answers were obtained without the MCDSS. The score increased to 76.25% after its use, which represents 60% enhancement. Both tests showed a good (even very good for the second one) enhancement with regard to the answers provided via use of the MCDSS.
- Unfortunately, the MCDSS prototype was not finished when the assessment campaign was carried out during the project. Consequently, it was not possible to assess the implementation corresponding to Specification 3 (identification of side effects associated with a recommended action). This will be of course done as soon as possible in the future. Nevertheless, the assessment results presented above suggest that these results will be also good. Indeed, finding all side effects between situations of interest (see Section 2.3, ANS-inter) may be a huge manual task as more than a 100 decision trees may be defined for a given cheese chain.

### 3.2. Technological efficiency aggregation assessment (Specification 4)

This assessment was conducted with a group of five technicians, all of whom were experts of a real cheesemaking process. It was focused on a set of three decision trees corresponding to three situations of interest (*Excessive dripping, Excessive acidification*, and *Excessive salting*). The five technicians provided individual assessments for 44 actions related to the three decision trees. Each action was assessed twice (88 assessments), with each assessment corresponding to two different production approaches: production approach 1 and production approach 2. Aggregated values were computed using the model presented in Section 2.2.1. In parallel, this group of experts collectively determined an assessment for each action without using the model. Computed assessments were compared to collective assessments. The associated data are available in Buche et al. (2022). Two assessments were considered to be in disagreement when there was a difference of at least two modalities (e.g., <no effect, moderately effective>, <very effective, not very effective>, etc.). The results presented in Table 1 show an error rate of around 5.7% (Total number of disagreements/Total number of actions), which is rather low. In practice, the method was considered relevant enough to compute an aggregated efficiency indicator associated with an action in a given decision tree. This aggregated indicator was discussed and validated collectively by the group of technicians during monthly meetings before being input in decision trees stored in the MCDSS knowledge base.

**Table 1:** Assessment of the efficiency aggregation method.

**Decision tree**	**Number of action assessments**	**Number of disagreements**
Excessive dripping	36	2
Excessive acidification	28	1
Excessive salting	24	2
Total	88	5

As already said above, the MCDSS prototype was not finished when the assessment campaign was carried out. Consequently, it was not possible to assess the implementation corresponding to Specification 4 about multicriteria filtering (identification of side effects associated with a recommended action).

## 4. Discussion

We discuss in this section the original contributions of the paper compared to the current state of the art, summarize and provide complementary information about key contributions and present some future directions of the research.

### 4.1. Comparison to the current state of the art

A lot of progress has been achieved in knowledge integration and multicriteria analysis methods and tools in the food science and technology field, yet they remain fragmented and incomplete (Aceves Lara et al., 2018; Thomopoulos et al., 2019). Different methods have been developed to gather scientific and technological knowledge and data for different purposes, but this information has only been general and focused solely on elementary processing operations, which do not take into account the entire processing operation. For example, Kansou et al. (2014) proposed a qualitative model of a unitary mixing operation using an expert system to predict the quality of wheat flour dough. Baudrit et al. (2015) modeled preharvest grape berry maturity—a critical characteristic for the wine industry—using expert knowledge and data and probabilistic graphical approaches. Belna et al. (2022) optimized microfiltration unit operation to integrate conflicting stakeholder objectives, such as maximizing product output quality while minimizing cost inputs and addressing environmental impacts. Baudrit et al. (2022) used data from scientific articles describing the entire milk microfiltration process including several unit operations in addition to the milk microfiltration step as skimming, heat treatment or storage. Those data are available in Buche et al. (2021). But the method presented in Baudrit et al. (2022) only proposes to learn a predictive model of the milk microfiltration unit operation in large-scale operational conditions including different membranes.

In a circular economy context, Belaud et al. (2022) proposed a decision-support system to rank alternative lignocellulosic waste transformation processes based on knowledge engineering tools to compile experimental data to assess potential environmental impacts. Munch et al. (2021) and Munch et al. (2022) combined ontology, probabilistic models and linked open data to generate, through a reverse engineering approach, agricultural wastes well-suited for processing biocomposites for food packaging. In both cases, ontological models facilitated analysis of the impact of the entire processing operation on the end-product quality or on indicators associated with the process, but the approaches required gathering of a substantial set of numerical experimental data to obtain good results. These approaches are unsuitable to achieve the objectives outlined in this paper because they are over-demanding in terms of obtaining sufficient numerical data to represent the entire range of collective knowledge at the level of a given food chain.

Fault tree analysis (FTA), which targets fault event risk assessment (Baig et al., 2013), may be compared to our approach. FTA enables computation of a level of risk represented by the occurrence probability of an undesired event. FTA also helps identify critical safety solutions to avoid the risk. For instance, in Pahasup-anan et al. (2021), the authors analyzed different situations that could trigger a dust explosion in an extruded food production facility. Kim et al. (2020) used FTA to assess the level of risk of four situations which could help determine the risk of microbial contamination of food by *E. coli*. A fault tree includes a root node representing the undesired event. The branches of the tree represent the explanatory scenarios, which may explain the fault event starting from basic events representing situations that would likely contribute to the overall fault defined in the roots. A whole fault tree could be considered as a set of scenarios associated with a probability of occurrence. Risk analysis is of course essential. However, FTA quantitative analysis requires collection of basic event occurrence measurements.

Our core ontology, which defines the decision tree structure, is comparable to the tree structure used in FTA as we also represent a decision tree linking explanatory situations to a given situation of interest. However, our objectives are quite different (see Section 2.1). Our original contribution compared to FTA consists of: (i) proposing a semantic decision tree representation using Semantic Web languages to enable easier open data linkage with other sources of information available on the Web; (ii) representing levers and associated technological actions to solve a situation of interest; (iii) representing the action efficiency based on individual experience; (iv) representing contextual criteria associated with recommendations to help filter recommendations in a multicriteria way; and (v) identifying possible side effects associated with the implementation of a recommendation. Moreover, FTA aims to estimate the probabilistic risk of failure, which requires the availability and collection of a substantial amount of numerical data, whereas our approach is based on collecting and representing the collective technical know-how available for a given domain (company, food chain, etc.).

Our ontological model may be compared with the COOK ontology (Ghrab et al., 2017), the Core Ontology of Organization Know-How and Knowing-That. In COOK, Know-How is defined as the capacity/disposition to perform an action. The COOK Know-How concept is similar to our Situation concept. COOK proposes a rich taxonomy to categorize different kinds of know-how (individual, collective, internal, external, crucial, etc.). From this viewpoint, we could consider that in the MCDSS the Situation concept is a specialization of CollectiveKnow-How. Our ontology enables us to represent complex interactions between Situations (conjunction, reinforcement, weakening) which is not possible in COOK. In COOK, the Knowing-That concept—a kind of belief—represents the relation between a proposition and a thinker. It assigns a truth value to the proposition. It is harder to compare this part of the COOK ontology to ours. Indeed, in our ontology, we implicitly consider that we represent a collective belief state, i.e., a COOK concept. On the other hand, we propose a more elaborate representation of the COOK Proposition concept as we represent expert reasoning using the notion of causality between situations, the efficiency of an action and associated contextual criteria. In conclusion, there are several similarities between both ontologies with a richer description of kinds of know-how in COOK and an explicit representation of expert reasoning in our ontology, which is not present in COOK. The part of our ontology which more or less corresponds to the Knowing-That concept part of COOK is more detailed because we have proposed a complete and operational MCDSS based on our ontology. Our ontology, like that of COOK, may be applied to any application domain based on know-how.

Compared to Buche et al. (2019), i.e., preliminary research that gave rise to the study described in this paper, several new contributions are proposed and assessed: (i) a model to aggregate action efficiency based on individual experience; (ii) an extended version of the ontology expressed in OWL, including the representation of action efficiency and associated information (key and complementary criteria); and (iii) the definition and implementation of the action view.

### 4.2. Key contributions

Here we showcased a new multicriteria decision-support system based on collective know-how in food chains to enhance food quality during the production process. This MCDSS is currently being used in production conditions in 13 AOP cheesemaking chain organizations involving professional stakeholders (cheese producers, experts working in technical centers) and professors from technological schools (ENILs), thereby comprising more than 60 users. Note that the model used to represent the decision trees may be refined in a flexible way until the level of detail sought by the expert is reached. This flexibility has been successfully used in the project as professors from technological schools have created generic cheese-making decision-trees. Operators introducing new chains in the project have tailored these generic decision trees to their specific cheese-making process.

Most of the MCDSS functionalities implemented to fulfill the specifications have been assessed, with promising results overall. All of the ontological model concepts are required to implement the MCDSS specifications. The choice to represent the causality relation between situations may be discussed. Indeed, a *HasCausality* semantic relation between two *Situation* nodes would have also been possible and simpler. However, as presented below in the perspectives of this work, it would be useful for advanced users conducting statistical analyses to be able to qualify the causality relation between situations in a future version of the MCDSS. By example, we would like to distinguish between types of sources, which contain the statistical analysis (internal study, bibliographical study). This metadatum should be associated with Causality nodes.

This justifies the modeling choice to set the stage for this future development. The comparison with and without the MCDSS was important to convince technicians of the MCDSS relevance. There are currently many obstacles to innovation, especially when it comes to know-how of which experts are sometimes afraid of losing (loss of employment, of power, etc.). Moreover, the direct use of mind maps without MCDSS assistance could be questioned. From our viewpoint the relevance is limited since the use of a simple mind map does not allow for three kinds of computerized analysis:

- The first corresponds to numerical aggregation of individual action efficiency assessments (see Specification 4). It provides an aggregated indicator, which is discussed and validated collectively by the technician team to determine the final action efficiency value, especially in the event of major disagreement. This value enriches the decision tree associated with a given situation of interest registered in the knowledge base.
- The second consists of the action multicriteria filtering mechanism generated by MCDSS queries, which reduces the list of candidate actions for a given situation of interest (see Specification 4).
- The third corresponds to the computing of complementary answers to enable assessment of the potential side effects with other actions and situations of interest (see Specification 3).

A typical use case of the MCDSS, which illustrates the relevance of those computerized analysis, is the following. A cheese maker has a problem of excessive salting. He/she queries the MCDSS on his/her phone using the *excessive salting* decision tree. First, using the Action view (see Figure 7), he/she selects the three corrective actions, which are very effective with a good/average agreement between experts of the chain. Clicking on the button identifying possible side effects (see Figure 8), he/she understands that using the *Brine duration* lever could be risky as four other defects may appear. Therefore, he/she navigates in the Explanatory view (see Figure 6) to compare the two remaining recommended actions (acidify or dilute the brine) will be able to verify if the analytical value associated with the situation *High brine density* corresponds to his/her actual situation to choose the action he/she will use.

Collected know-how consistency checking is consolidated and enriched throughout the workflow presented in Figure 1. First note that Buche et al. (2019) proposed a method that enables collective mind mapping dedicated to this MCDSS. Consequently, decision trees which are outcomes of this collective mind mapping activity are already validated as they contain the consolidated knowledge of the food chain experts resulting from collective discussion. Secondly, it is possible to verify that recommended actions are relevant by checking the technicians' feedbacks (Specification 4) after implementation of the recommendations. If the actions are good, then the recommendation remains valid. Conversely, further investigation may be required to understand why a recommended action failed. Thirdly, criteria associated with action efficiency assessments may contain information to verify action's relevance. For instance, an advanced chain has defined two criteria using the MCDSS: (1) StatisticalResults (yes/no), meaning that statistical results validating the recommendation have been obtained in the food chain; (2) BibliographicalResults (yes/no), meaning that results published in a scientific paper have validated the recommendation. In both cases, a link to a complementary website may be embedded in the decision tree branch to provide more information. Fourthly, it is possible to determine if certain suggestions delivered by the MCDSS were not executed. Indeed, this kind of action may be identified if the *Never* modality is associated with the “*number of action implementations*” information (*hasForObservations* property associated with the *Efficiency* concept). Explanations may be provided by analyzing values associated with the *ConsequenceCriteria* associated with the *Efficiency* concept. For instance, a given action has never been executed because it could generate a sanitary risk or be costly to implement. Fifthly, the collective mind mapping activity conducted to create and maintain decision trees may identify knowledge gaps. This means that in a given situation the experts may not know which action to recommend or may disagree on the action to recommend. Sometimes, they may know the action to recommend to solve a given situation of interest, without being able to explain why. In all of those cases, new experiments may be conducted to unlock knowledge gaps. Learning improvement in novel knowledge gap cases is a natural outcome of the collective mind mapping activity, which is the first step of the MCDSS workflow process.

In terms of upscaling, more advanced chains manage around a 100 decision trees and they will certainly increase to several hundreds. But big data are not involved and no scalability problems in terms of volumes should arise because we only represent expert knowledge. The problem would arise if we were to seek to represent the numerical experimental data so as to be able to create/assess this expert knowledge. But this is beyond the scope of this work.

## 5. Conclusion and perspectives

The perspectives of this project are numerous. In AOP cheesemaking chain organizations, the priority has been toward recommendations to correct organoleptic defects. But in the future the method will enable us to create decision trees to recommend actions to solve food safety problems or to achieve a given food quality. Moreover, we will extend know-how representation to the upstream part of the chain, including milk production. New methodological challenges will be tackled to take spatio-temporal knowledge representation into account. Advanced users who conduct statistical analyses may like to be able to qualify the causality relation between situations in a future version of the MCDSS. This extension will be easy to design thanks to the choice made in the ontology model to represent causality relations. Another prospect will be to take new MCDSS sustainability criteria into account. We will focus specifically on the environmental impact of cheese production. Using Semantic Web languages to implement the knowledge base will facilitate interoperability management with new sources of information that are also managed with those languages (Pénicaud et al., 2019; Cortesi et al., 2022a,b).

This MCDSS is a generic tool, which could potentially be used in different food and bio-product chains. Encouraging preliminary tests, as reported in Buche et al. (2019), have been conducted in the cereal (couscous) and dairy sectors (instant milk powder). Consequently, new dissemination activities will be conducted in the future.

## Data availability statement

The datasets presented in this study can be found in online repositories. The names of the repository/repositories and accession number(s) can be found at: https://doi.org/10.57745/SEJP1B.

## Ethics statement

Ethical approval was not required for the study involving human participants in accordance with the local legislation and institutional requirements. Written informed consent to participate in this study was not required from the participants in accordance with the national legislation and the institutional requirements.

## Author contributions

PB, SD, JCu, AO, and JCo contributed to conception and design of the study. JCu and AO organized the database. PB and JCo performed the statistical analysis. PB wrote the first draft of the manuscript. PB and SD wrote sections of the manuscript. All authors contributed to manuscript revision, read, and approved the submitted version.

## Footnotes

^1^The actual numerical values have been anonymized to avoid recognition of the cheese chain.
